# Optical screw-wrench for microassembly

**DOI:** 10.1038/micronano.2016.83

**Published:** 2017-02-27

**Authors:** Jannis Köhler, Sarah Isabelle Ksouri, Cemal Esen, Andreas Ostendorf

**Affiliations:** 1 Applied Laser Technologies, Ruhr-Universität Bochum, Universitätsstraße 150, Bochum 44801, Germany

**Keywords:** holographic optical tweezer, microassembly, microfabrication, microfluidics, optical assembly, screw connection, screw-wrench, two-photon polymerization

## Abstract

For future micro- and nanotechnologies, the manufacturing of miniaturized, functionalized, and integrated devices is indispensable. In this paper, an assembly technique based on a bottom-up strategy that enables the manufacturing of complex microsystems using only optical methods is presented. A screw connection is transferred to the micrometer range and used to assemble screw- and nut-shaped microcomponents. Micro-stereolithography is performed by means of two-photon polymerization, and microstructures are fabricated and subsequently trapped, moved, and screwed together using optical forces in a holographic optical tweezer set-up. The design and construction of interlocking microcomponents and the verification of a stable and releasable joint form the main focus of this paper. The assembly technique is also applied to a microfluidic system to enable the pumping or intermixing of fluids on a microfluidic chip. This strategy not only enables the assembly of microcomponents but also the combination of different materials and features to form complex hybrid microsystems.

## Introduction

Miniaturization and multifunction integration are ongoing trends in microsystem technologies. In microfluidic systems, for example, multiparallel analyses and diagnostics of reagents can be accomplished on only a single chip, for example, a laboratory-on-a-chip or a micro total analysis system. Due to the low amount of liquid in the samples, rapid reaction times and low waste generation can be achieved and are thus highly advantageous features of such systems. To manufacture such devices with functional integration at the micro- or nanometer scale, micro-stereolithography processes can be utilized. In recent years, two-photon polymerization (2PP or TPP) has emerged as a powerful tool for the fabrication of three-dimensional (3D) microstructures^1^. The high resolution and high degree of design flexibility conferred by 2PP means that it can be applied to different microfluidic applications in which liquid fluids need to be transported, mixed, or controlled by means of valves or switches^2^. Furthermore, by using photosensitive resins that contain the appropriate nanomaterials, different functions can be incorporated into the 2PP-fabricated microstructure^3,4^. For example, one could potentially make the microstructure electrically conductive, or have a refractive index that varies as a function of position, or make the microstructure chemically functionalized. In addition, magnetic microstructures can be manufactured and actuated using an outer magnetic field. This feature enables the generation of magnetic microturbines and -rotors to displace the surrounding fluid in a microchannel for pumping or mixing applications^5^.

However, the combination of different materials and multiple functions within a microstructure produced by 2PP remains a challenge. Nevertheless, bottom-up techniques can be used to assemble microcomponents or building blocks with different properties to form complex hybrid microsystems. Mechanical microgrippers, for instance, can pick up, position, and place different microcomponents in a precise manner to the correct locations. However, adhesion forces overcome gravity forces at the micrometer scale and make it difficult to release the microcomponents of the gripper^6,7^. Thus, the assembly techniques presented here are based on an optical gripper. Utilizing the optical forces in optical tweezers (OTs) provides contactless trapping, moving, and manipulation of structures at the micrometer scale; thus, adherence is not an issue^8^.

Čižmár *et al.*^9^ used an OT to trap and position a large number of microparticles in a liquid environment. In addition, complex 2PP-fabricated microcomponents could be assembled on a planar surface^10^. Therefore, automatic computer-aided detection, orientation, and positioning of components can be accomplished. However, there is no stable connection between the microcomponents; thus, when the laser is switched off, the microcomponents drift apart due to Brownian motion.

Ghadiri *et al.*^11^ used biomolecules with high affinities applied as particle coatings to manufacture stable planar and 3D microstructures. The complementary particles were optically trapped, positioned, and joined by bringing them into contact. Furthermore, particles with varied optical properties have been assembled to build hybrid microstructures. Although this biochemical coating does not allow disassembly of the microstructures, approaches have been developed utilizing (re-)programmable DNA–protein interactions to enable binding and release^12^. Nevertheless, this method has not been studied in relation to microassembly using OTs. Other groups have used thermal and photothermal effects^13,14^ or photopolymerization^15,16^ to stably join microparticles. However, these assembly techniques also do not provide a releasable connection. Once the microstructures are joined together, it is not possible to release them without destroying the components.

Interlocking or form-fit connections can be applied to assemble and disassemble 2PP-fabricated microcomponents^6,17,18^. Careful design and construction can enable the manufacturing of microcomponents that are complementary, fit together, and have a stable and releasable connection. In addition, this technique would enable the assembly of multiple materials with different functions. However, in microchannels of microfluidic systems, for instance, where high fluid flows occur, even these assembly techniques are not sufficiently stable.

The main aim of this paper is to present and investigate an all-optical approach for a stable and releasable connection between 2PP-fabricated microcomponents. More precisely, a screw connection is transferred to the micrometer range and used to assemble screw- and nut-shaped microcomponents utilizing optical forces. Accordingly, the OT is used as an optical screw-wrench. This technique could be beneficial for a bottom-up approach to assemble complex microsystems. To demonstrate the functionality of this approach, the screw connection is applied to a microfluidic device. A micro-rotor is assembled and actuated, and the screw connection is used to fix and hold the rotor on its rotational axis.

## Materials and methods

### Fabrication of microcomponents

For precise 3D microfabrication, 2PP has been established as a rapid prototyping technology with submicrometer resolution^19^. By tightly focusing ultrashort laser pulses into a photosensitive material, a chemical reaction is triggered and polymerization occurs. Arbitrary structures are fabricated by scanning the laser beam through the material layer by layer with a 2D galvo mirror system. The resolution depends on the optical configuration, for example, the numerical aperture (NA) of the focusing objective, the scanning speed, the laser power, and the properties of the photosensitive material. Resolutions of less than 100 nm have been reported^1,20^.

**Figure 1:**
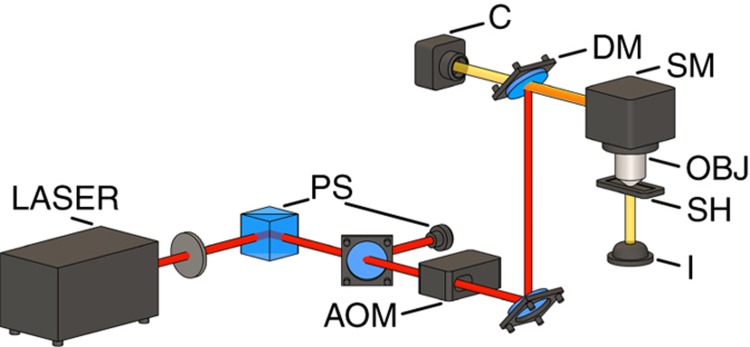
Schematic depiction of the experimental set-up used for 2PP. AOM, acousto-optic modulator; C, camera; DM, dichroic mirror; I, white light illumination; OBJ, microscope objective; PS, power setting; SH, sample holder; SM, scanning galvo mirror system.

The experimental 2PP set-up is illustrated in Figure 1. To reach sufficient radiant flux density, a mode-locked titanium-sapphire laser (Tsunami, Spectra Physics, Stahnsdorf, Germany) with a pulse width of 90 fs, a repetition rate of 82 MHz, and a central wavelength of 780 nm is used. The laser power can be adjusted using a motorized *λ*/2 waveplate in combination with a polarizing beam splitter. The power setting is controlled via a photodiode. An acousto-optic modulator (AOM) enables rapid operation and is utilized as a beam shutter. A scanning galvo mirror system (Hurryscan II, Scanlab, Puchheim, Germany) is used for beam steering. Subsequently, the laser beam is tightly focused, using an oil-immersed objective with an NA of 1.4, through a coverslip into a drop of negative photoresist (Femtobond 4B, Laser Zentrum Hannover e.V., Hannover, Germany). This coverslip is mounted on a sample holder, which can be moved axially with a motorized stage (Wafer Max Z, Aerotech Inc., Pittsburgh, PA, USA). Thus, 3D microstructures can be fabricated by illuminating the photoresist line by line. The process can be monitored using integrated white light illumination (I) and a camera (C). As processing parameters, a scanning speed of 1.6 mm s^−1^, an average laser power of 20 mW (measured after the AOM), and a line distance of 0.1 μm were selected. After the exposure of the desired microstructures, the non-polymerized regions are washed out by utilizing a suitable developer (OrmoDev, Micro Resist Technology GmbH, Berlin, Germany) with 0.2% of Tween^®^ 20.

### Optical assembly

In 1970, Ashkin^21^ was able to observe the effects of radiation pressure on dielectric microparticles. Subsequently, the trapping and moving of a spherical particle in all three dimensions by means of a tightly focused laser beam was demonstrated^22^. The optical force acting on the particle depends on the momentum transfer between the photons and matter and is proportional to the laser power. Sophisticated OTs enable the simultaneous manipulation of multiple objects using a single laser source. With the use of a spatial light modulator (SLM), multiple trapping spots can be originated by computer-generated phase patterns^23^. Furthermore, these spots can be moved in all three dimensions individually. These SLM-based optical grippers are commonly referred to as holographic OTs (HOTs).

**Figure 2:**
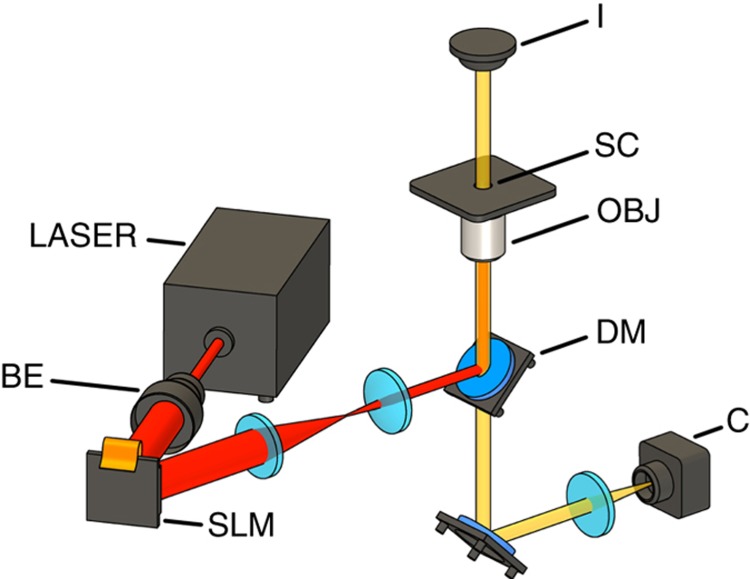
Schematic depiction of the experimental set-up used for the HOT. BE, beam expander; C, camera; DM, dichroic mirror; I, white light illumination; OBJ, microscope objective; SC, sample chamber; SLM, spatial light modulator.

The set-up of the HOT used in the experiments is illustrated in Figure 2. The continuous wave fiber laser (Gevel, Manlight, Lannion, France) with a wavelength of 1064 nm, the phase modulating reflective SLM (PLUTO-NIR, Holoeye, Berlin, Germany), and the microscope objective with an NA of 1.45 represent the main components. The SLM is integrated to trap and control complex microstructures by generating multiple trapping spots. The laser light is focused into the sample chamber, which is typically a small microfluidic chamber with dispersed microparticles or -components. The sample chamber is mounted directly over the OBJ and is controlled using a motorized positioning stage. Hence, relative movement in all three dimensions between the objective and the sample chamber is achievable. Trapping and moving of the microcomponents is monitored by an integrated CMOS-camera (C) and white light illumination (I).

### Design and construction of microcomponents

To verify the functionality of a screw connection in the micrometer range, screw- and nut-shaped microstructures were fabricated and screwed together with the use of optical forces. Therefore, several design and construction requirements are essential, and they will be discussed in the following paragraphs.

**Figure 3:**
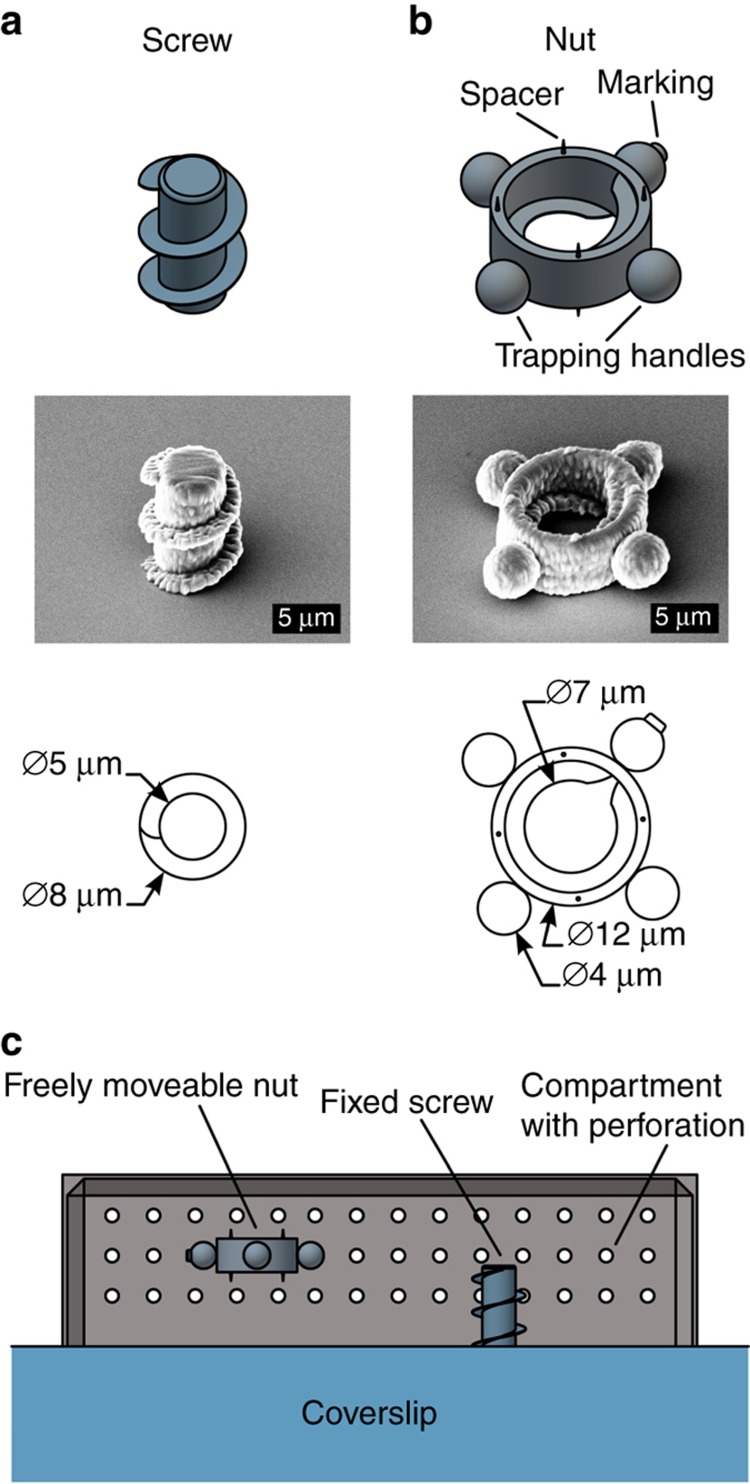
Schematic depictions, SEM images, and technical drawings of the screw (**a**) and nut (**b**) are shown. (**c**) To prevent the nut from washing away during the development process, the microstructures are enveloped by a compartment structure, which is illustrated by the section view. SEM, scanning electron microscope.

The exposure process for the screw starts inside the coverslip to ensure a stable joint at the interface. Thus, the screw is fixed on the coverslip. The microcomponent is manufactured according to the dimension specifications, as shown in Figure 3a. The pitch is 5 μm. The nut, as the complementary part to the screw, must be freely movable to enable assembly. Thus, the nut is fabricated inside the photopolymer and has no contact with the coverslip. Different approaches for the optical trapping and movement of complex microstructures with arbitrary shapes were studied, for example, multiple trapping spots^24^, controlled torque by aligning linear polarized light^25,26^, or customized beam shapes^27^. In this particular case, four spheres are attached to the nut and have sufficient ease of handling to generate the four trapping spots (Figure 3b). Optical forces can act on these trapping handles and provide stable movement and rotation of the nut in all dimensions. The spheres are 4 μm in diameter. In addition, one handle is marked with a small cylindrical structure to enable monitoring of the nut orientation under the microscope. To reduce surface contact with the coverslip and reduce adhesion effects, four spacers are attached to both sides of the nut.

Because the nut is freely movable, it can easily be washed away from its generated position during the development process. The time for relocalization of the produced nut was significantly shortened by using a compartment structure^28,29^. This compartment structure envelopes the nut and prevents it from being washed away during the development process. A section view of the compartment structure is shown in Figure 3c; it is fixed on the coverslip and has uniform perforated holes with diameters of ~1 μm. Through these holes, the development process can be accomplished. In comparison to the screw and nut, the compartment structure does not need to be produced with a high level of detail. To reduce the processing time, the line distance can be adjusted to 0.5 μm.

After the exposure process of all three components is complete, the coverslip is sealed to an aluminum part with a circular hole of 10 mm, which forms the sample chamber. The developer is filled into the hole and the sample chamber is sealed with another coverslip on top of it.

## Results

### Releasable screw connection

**Figure 4:**
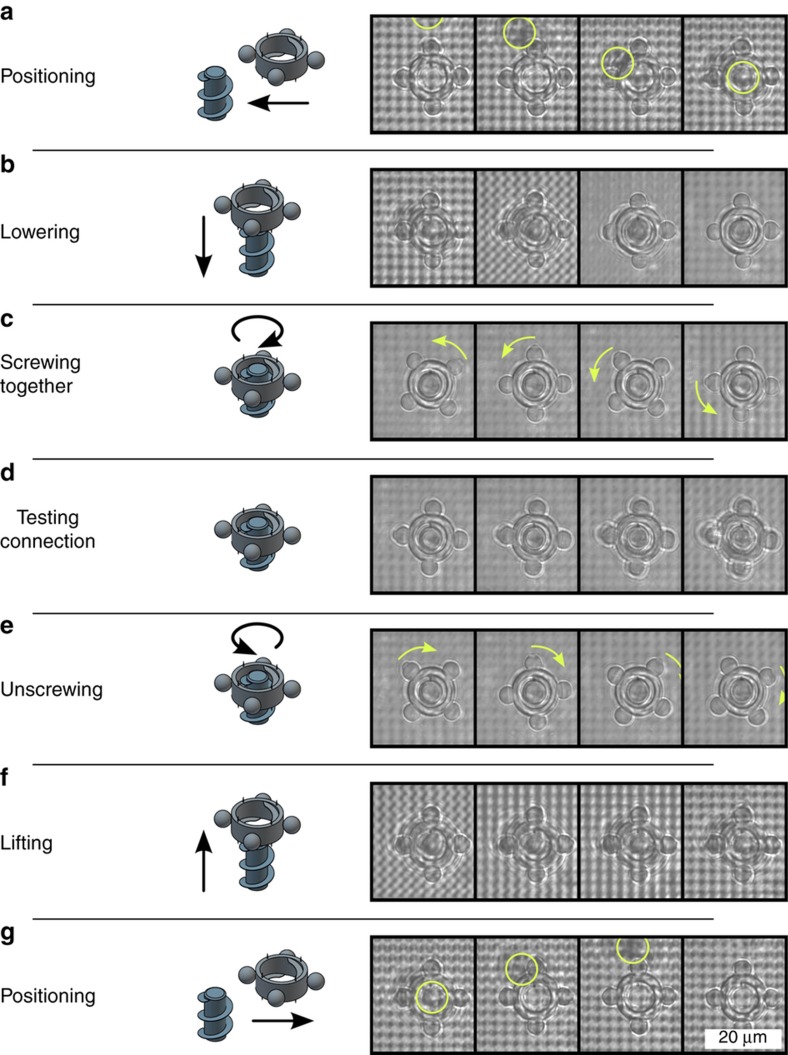
The sequence of images (**a**–**g**) show assembly and disassembly of the nut and screw with the use of the optical screw-wrench (inserted rings and arrows indicate the screw and rotation directions, respectively). A releasable screw connection can be realized.

The sequence of microscope images in Figure 4 demonstrates a releasable connection of the screw- and nut-shaped microstructures. By generating four optical trapping spots with a total laser power of 174 mW (measured after the SLM), the nut can be trapped at its four handles, moved, and positioned over the screw, which is highlighted by the inserted rings (a). Next, the nut is lowered axially until it touches the top of the screw (b). It should be noted that the camera is mounted under the microscope objective and that the images are captured from below through the coverslip. Consequently, the microscope images are mirrored. In addition, it is notable that as the compartment structure and its perforation become sharper the higher the nut is lifted. In addition, this can be used to estimate the axial height of the trapping spots. While rotating the trapping spots counterclockwise, the nut follows, as indicated by the marked trapping handle and the inserted arrows, and a connection is established (c). During rotation, the axial position must be adjusted. After screwing the components together, the connection can be tested by raising the axial position (d). It can be demonstrated that the contour of the nut becomes blurred, which indicates that it cannot be lifted by optical forces and a stable connection can be assumed. The nut can be unscrewed by rotating it clockwise, as indicated by the inserted arrows (e). After disassembly, the nut can be released (f) and positioned beside the screw (g).

**Figure 5:**
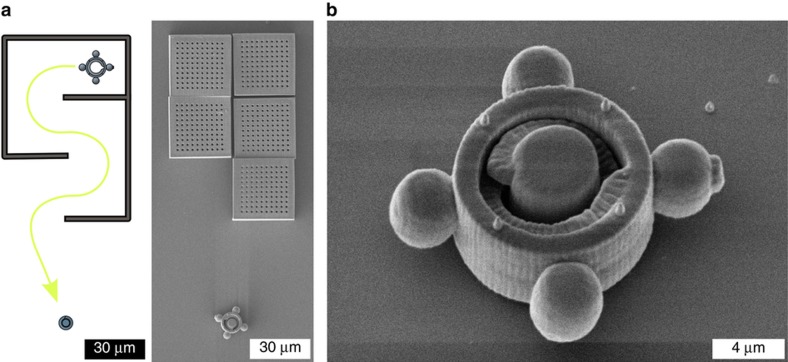
(**a**) Schematic depiction of the maze-like compartment. Using optical forces, the nut can be guided outside and assembled with the fixed screw. (**b**) The SEM image shows a stable screw connection of the microcomponents.

To analyze the screw connection with high resolution by scanning electron microscope (SEM), a different compartment structure is crucial. The compartment structure described limits the visibility inside, and the screw connection cannot be studied. Therefore, the compartment must be redesigned as a maze-like structure (Figure 5a). The nut is fabricated inside and the screw is positioned outside in front of the compartment structure. Thus, the nut is prevented from washing away during the development process and, at the same time, it can be trapped and guided outside to the screw. After screwing the components together, the sample can be dried, sputtered, and investigated using SEM. Figure 5b shows a stable connection of the microcomponents with a high level of detail.

### Micro-rotor assembly and actuation

The rotational motion of microstructures in microchannels can be used to mix different liquid samples or induce directed flow^30^. In the following paragraphs, the optical assembly technique presented is applied to such a microfluidic application. A micro-rotor is manufactured by 2PP and optically assembled and actuated. The screw connection is used to hold the rotor on its rotational axis. In general, the flow fields generated in a microchannel can also be investigated using HOTs^31^.

**Figure 6:**
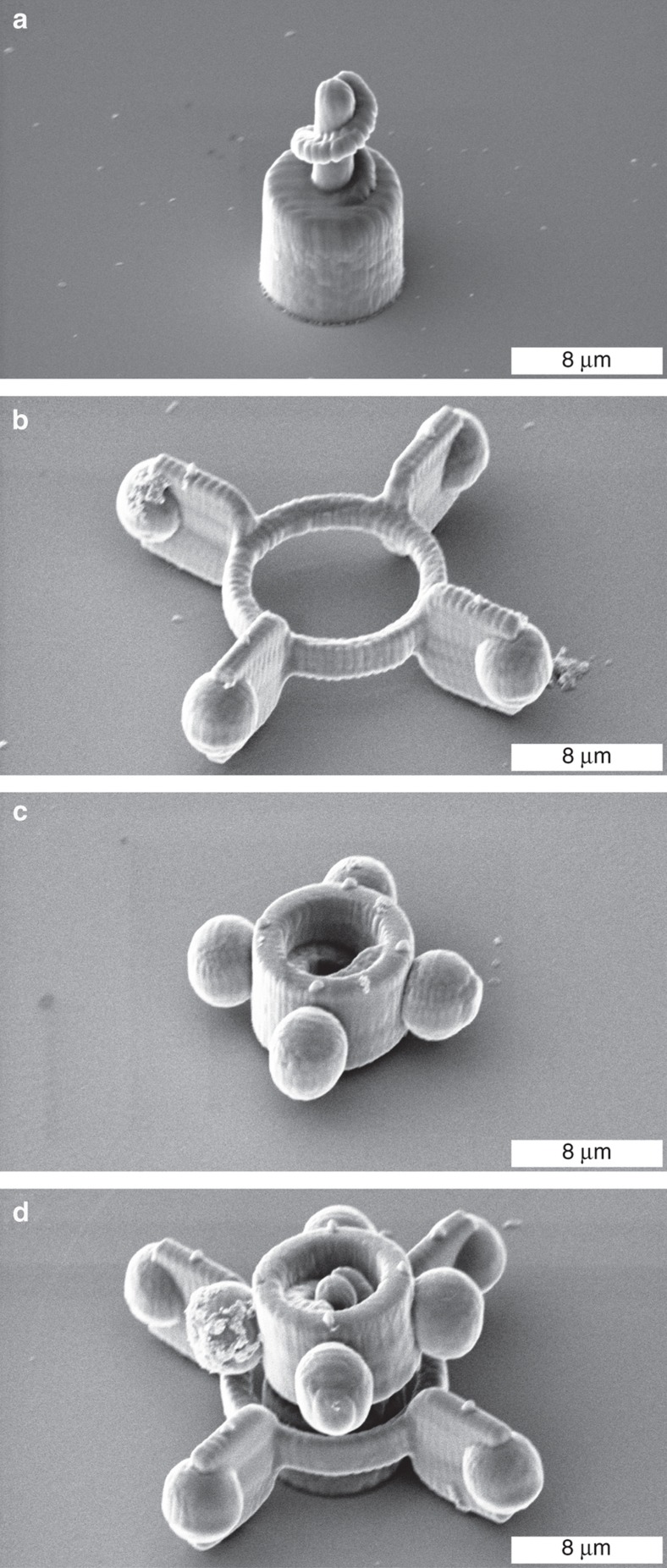
SEM images of the three microcomponents for assembling a micro-rotor are shown at a tilt angle of 45°. The rotational axis (**a**) is fixed on the coverslip, whereas the rotor (**b**) and the nut (**c**) are freely movable and can be assembled with it. The completed microsystem for use as a microfluidic application is shown in (**d**).

SEM images of the three microcomponents used are shown in Figure 6. The axis (a) is fixed on the coverslip. The axis can be divided into a cylinder at the bottom, which is used as the rotational axis and a screw thread on top. The freely movable rotor (b) can be moved optically and positioned due to the four trapping handles. Here, a simple rotor geometry with four blades to displace the surrounding fluid during the rotation is selected. However, with the use of the 2PP technique, the fabrication of different rotor types would be feasible. The number and the shape of the blades could easily be adjusted as desired for different requirements in microfluidic applications. As mentioned previously, it is also possible to manufacture magnetic rotors. For the rotor presented, four spacers were utilized to reduce the surface contact and effects of adhesion. After assembling the rotor and its rotational axis, the rotor can be fixed in position by screwing the nut (c) on top of it. One of the four trapping handles of the nut is marked for the purpose of monitoring the orientation. In addition, four spacers are attached to each side. Figure 6d shows the assembled micro-rotor under SEM. It can be demonstrated that there is a stable connection, and that the nut prevents the rotor from being washed away and holds it in the desired position.

**Figure 7:**
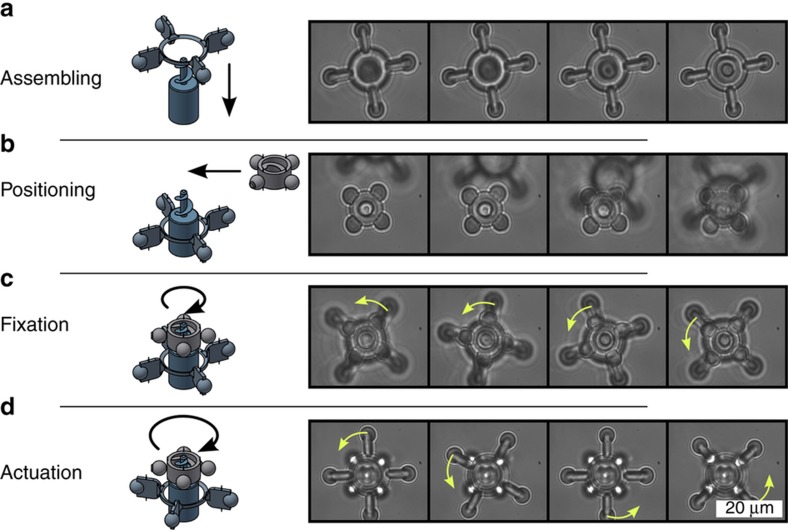
The sequence of microscope images (**a**–**c**) shows the assembly steps of the micro-rotor and the fixation due to the screw connection. After assembly, the rotor can be actuated (**d**) using optical forces (inserted arrows indicate the direction of rotation).

The micro-rotor can be rotated in a liquid environment after the assembly and screwing process, as presented in Figure 7. First, the rotor must be trapped and positioned over the axis. Again, a total laser power of 174 mW is used. After positioning, the rotor can be assembled along with the rotational axis by reducing the axial height (a). To prevent the rotor from pulling away or tilting due to the flow of liquid in the subsequent application as a micromixer or -pump, the screw connection can be utilized. In the process, the nut must be positioned (b), lowered, and screwed (c) on the axis. Now, the rotor is held in position. Actuation of the rotor can also be performed using optical forces (d).

There are different optical approaches for applying torque to a microstructure^32–34^. To demonstrate the functionality of the micro-rotor, in this work, the four trapping spots, which were also used for assembly, are rotated and the rotor follows. A maximum rotational speed of ~25 rpm can be achieved in both rotational directions. Compared with other optically driven micropumps in the literature, for example, Maruo *et al.*^35^, this rotational speed is relatively low. However, it is assumed that this rotational speed is sufficient for microfluidic systems, where low flow rates are required. To achieve higher rotating speeds, a higher laser power can be used or the optical traps can be optimized to increase the optical forces^36^.

## Discussion

To improve devices in microsystem technologies in terms of functional density or to develop novel features, miniaturization and integration are key. In transferring a screw connection to the micrometer scale, a unique bottom-up technique for assembling complex microsystems is achieved. Moreover, in this paper, a stable and releasable connection is demonstrated, which is achieved using only optical techniques.

First, the requirements for trapping and moving 2PP-fabricated microstructures were discussed with regard to the design and construction. Next, screw- and nut-shaped microcomponents were assembled using optical forces as an optical screw-wrench. A stable and releasable screw connection was presented, which would offer the integration of different functions into microsystems due to the combination and connection of different materials. After demonstrating the functionality of the screw connection, it was applied to a microfluidic application. A microsystem composed of a rotational axis, a rotor with four blades, and a nut was assembled. Due to the screw connection, the rotor was fixed on its rotational axis. It is assumed that the micro-rotor is sufficient for displacing a low number of liquid samples and, therefore, could be useful for pumping and mixing applications. However, the mode of driving must be studied and reviewed to improve the rotational speed of the rotor.

A screw connection at the micrometer scale provides further benefits for different applications. To analyze and study single biological probes, such as cells or bacteria, it is often necessary to fix them on a coverslip without the use of toxic adhesives. Utilizing the screw connection would provide a minimally invasive physical fixation in a liquid environment. In addition, the sample could be released if desired. Furthermore, similar to gears and transmissions, miniaturized screws and threads provide force and motion transmission for applications in microrobotics and micromechanics. Using the approach presented, assembly of such mechanically stable systems and easy disassembly would be achievable.
